# *Rheb1*-Deficient Neutrophils Promote Hematopoietic Stem/Progenitor Cell Proliferation *via* Mesenchymal Stem Cells

**DOI:** 10.3389/fcell.2021.650599

**Published:** 2021-05-27

**Authors:** Juan Gao, Shuaibing Hou, Shengnan Yuan, Yuxia Wang, Yanan Gao, Xiaolu Sun, Weili Wang, Yajing Chu, Yuan Zhou, Xiaoming Feng, Hongbo R. Luo, Tao Cheng, Jun Shi, Weiping Yuan, Xiaomin Wang

**Affiliations:** ^1^State Key Laboratory of Experimental Hematology, National Clinical Research Center for Blood Diseases, Institute of Hematology and Blood Diseases Hospital, Chinese Academy of Medical Sciences & Peking Union Medical College, Tianjin, China; ^2^Tianjin Key Laboratory of Ophthalmology and Visual Science, Tianjin Eye Hospital, Clinical College of Ophthalmology, Tianjin Eye Institute, Tianjin Medical University, Nankai University Affiliated Eye Hospital, Tianjin, China; ^3^Department of Pathology, Tianjin Medical University Cancer Institute and Hospital, Tianjin Medical University, Tianjin, China; ^4^Department of Pathology, Dana-Farber/Harvard Cancer Center, Harvard Medical School, Boston, MA, United States; ^5^Department of Neuro-Oncology, Cancer Center, Beijing Tiantan Hospital, Capital Medical University, Beijing, China

**Keywords:** *Rheb1*-deficient neutrophils, hematopoietic stem/progenitor cell, proliferation, mesenchymal stem cells, IL-6

## Abstract

Myeloid cells have been identified as hematopoietic stem cell (HSC)-regulating cells. However, the mechanisms by which myeloid cells regulate the function of HSCs are not fully defined. Our previous study indicated that the HSCs are over-expanded in *Vav1-Cre;Rheb1^*fl/fl*^* mice. Here, using *in vivo* and *in vitro* models, we found that *Rheb1*-deficient neutrophils remodeled the bone marrow environment and induced expansion of HSCs *in vivo*. Further studies showed that loss of Rheb1 impaired neutrophils’ ability to secrete IL-6, led mesenchymal stem cells (MSCs) to produce more SCF, and promote HSC proliferation. We further found that IL-6 suppressed SCF mRNA expression in human MSCs. Interesting, the high level of IL-6 was also related with poor survival of chronic myeloid leukemia (CML) patients, and higher expression of IL-6 in CML cells is associated with the lower expression of SCF in MSCs in patients. Our studies suggested that blocking IL-6 signaling pathway might stimulate MSCs to secrete more SCF, and to support hematopoietic stem/progenitor cells proliferation.

## Introduction

Hematopoietic stem cells (HSCs) play an important role in the regulation of hematopoietic homeostasis. They can self-renew and differentiate into all cell types of the hematopoietic system according to proliferative or stress cues throughout life (Feng et al., 2008; Rathinam and Flavell, 2008). This process requires fine regulation by intra- and extracellular signaling in the bone marrow microenvironment. HSCs primarily reside in the bone marrow cavity (or niche), where they interact with a variety of cell types, including perivascular cells, osteoblast cells (OBCs) and mesenchymal stem cells (MSCs). HSCs directly adhere to mesenchymal cells and possibly to osteoblasts in the bone marrow stroma that express important regulatory molecules including stem cell factor (SCF) and C-X-C motif chemokine 12 (CXCL-12) (Frenette et al., 2013; Greenbaum et al., 2013). In addition, differentiated hematopoietic cells have been proposed to regulate HSCs. For example, macrophages have been shown to indirectly promote the retention of HSCs by regulating mesenchymal cells and osteoblasts (Chow et al., 2011). Megakaryocytes (MK) are physically associated with HSCs in the bone marrow. MK ablation leads to the activation of quiescent HSCs and increases proliferation of HSCs (Meng et al., 2014). Neutrophils can produce ROS to stimulate the proliferation of myeloid progenitors (Kwak et al., 2015). These studies suggested that the hematopoietic cells and niche cells interact with HSCs, regulate HSCs division and proliferation, and maintain hematopoietic system balance *in vivo*.

It has been known that the change of normal hematopoietic niche to a hostile HSC growth environment is the tipping point for the development of myelodysplastic syndromes (MDS) and leukemia (Raaijmakers, 2012). For example, study has shown that leukemia cells could secrete more proinflammatory cytokines and establish a feedback loop that drive over-expansion of immature myeloid cells and chronic myeloid leukemia (CML) development (Reynaud et al., 2011). Additionally, leukemia cells could also stimulate MSCs to differentiate into OBCs to support LSC proliferation (Schepers et al., 2013), while MSCs may accelerate abnormal HSCs over-proliferation through secreting more SDF-1 in myeloproliferative neoplasm (MPN) patients (Arranz et al., 2014).

mTOR is a serine/threonine protein kinase that responds to multiple signals and maintains homeostasis. Increased or decreased mTORC1 activity can alter HSC function and cause hematological disorders (Wang et al., 2016a). Rheb1 acts as a key activator of mTOR to play vital roles in maintaining proper hematopoiesis and myeloid differentiation (Aspuria and Tamanoi, 2004). Previously, we reported that *Rheb1*-deficient mice showed increased phenotypic HSCs, immature neutrophils in bone marrow, and splenomegaly, which are reminiscent of the hematopoiesis seen in MPNs (Wang et al., 2018). Meanwhile, *Rheb1* deficiency inhibits the development of macrophages and neutrophils, thus impairing their phagocytic ability (Wang et al., 2016b). Rheb1 cooperated with MLL-AF9 to promote acute myeloid leukemia progression, and deletion of *Rheb1* in the MLL-AF9 acute myeloid leukemia mouse model prolonged the survival of mice by inhibiting the mTORC1 signaling pathway (Gao et al., 2016).

Interestingly, our study found that the absolute number of HSCs were increased in *Vav1-Cre;Rheb1^*fl/fl*^* mice, while HSCs were not over expanded under *in vitro* culture conditions (Wang et al., 2018). This suggested that the expansion of these HSPCs in the bone marrow of *Vav1-Cre;Rheb1^*fl/fl*^* mice might be caused by additional extrinsic factors rather than intrinsic factors. Here in this study, we found that loss of *Rheb1* impaired neutrophils ability to secrete IL-6, and this in turn stimulated MSCs to produce more SCF, leading to HSPCs over proliferation.

## Materials and Methods

### Mice and Genotyping

*Vav1-Cre* mice (JAX stock #008610, background CD45.2) were purchased from Jackson Lab. *Rheb1^*fl/fl*^* mice (background CD45.2) were kindly provided by Dr. Bo Xiao (Zou et al., 2011). The mice were crossed with *B6.SJL* mice (background CD45.1) and were backcrossed, respectively, to generate CD45.1 congenital *Vav1-Cre* mice and *Rheb1^*fl/fl*^* mice (Supplementary Figure 1A). Then the *Vav1-Cre* mice and *Rheb1^*fl/fl*^* mice were crossed to generated mice in which *Rheb1* was specifically deleted in the hematopoietic system (*Vav1-Cre;Rheb1^*fl/fl*^* or *Rheb1*^Δ/Δ^, CD45.1). C57BL/6 mice (6–8 weeks old, CD45.2) were the recipient mice in transplantation experiments. Mice were maintained at the specific pathogen-free (SPF) animal facility of the State Key Laboratory of Experimental Hematology (SKLEH). All animal surgeries were approved by the Institutional Animal Care and Use Committee (IACUC), Institute of Hematology and Blood Diseases Hospital, CAMS/PUMC. All efforts were made to minimize mouse suffering.

### Flow Cytometry Analysis

A 15 μl peripheral blood (PB) sample was obtained from either the tail vein or retroorbital bleeding and diluted with PBE (PBS with 2% fetal bovine serum and 2 mM EDTA). Before staining, ammonium chloride-potassium bicarbonate was used to lyse the red blood cells (RBCs). Bone marrow (BM) cells were flushed out from tibias, femurs and ilia with PBE. The cells were stained with the following antibodies: anti-mouse CD3 biotin, anti-mouse CD4 biotin, anti-mouse CD8a biotin, anti-mouse TER-119 biotin, anti-human/mouse CD45R (B220) biotin, anti-mouse CD11b BIOTIN, STREPTAVIDIN APC-Cy7, anti-mouse CD34 FITC, anti-mouse CD117 (c-Kit) APC, anti-mouse Ly-6A/E (Sca-1) PE-Cyanine7, anti-mouse CD45.2 PE, and anti-mouse CD45.1 Percp-Cy5.5 for HSPCs or anti-mouse CD45.1 FITC, anti-mouse CD45.2 PE, anti-mouse CD11b APC, and anti-mouse Ly-6G (Gr-1) PE-Cyanine7 for neutrophils. All antibodies were purchased from either eBioscience or Invitrogen (United States). The detail information for the antibodies was listed in the Supplementary Table 1. The samples were analyzed with a BD Canto II flow cytometer, more than 10,000 cells were collected and the results were analyzed with FlowJo software.

### Isolation of Neutrophils From Bone Marrow

Percoll-based (GE Healthcare, 17144003, Little Chalfont, United Kingdom) density gradient centrifugation was used for the purification of neutrophils from bone marrow cells (Swamydas et al., 2015). A “100% Percoll” solution is generated by adding 5 ml of 10X HBSS (Gibco, 14065056, United States) to 45 ml of Percoll. Percoll dilutions of 52, 62, and 76% were generated from the “100% Percoll” solution using 1X HBSS-EDTA (Thermo Fisher Scientific, 14025092, United States). The 76, 62, and 52% Percoll separation solutions were successively added to a 15 ml centrifuge tube (avoiding mixing of the three concentrations of Percoll separation solutions). The bone marrow cell suspension was overlaid on the Percoll separation layer, followed by centrifugation for 30 min at 2,800 rpm (1,420 g) at room temperature without braking. Cells were harvested from the 76 and 62% Percoll interface and washed twice with 1X HBSS buffer. 3 ml of Histopaque-1119 (Sigma-Aldrich, 11191, United States) was added to a 15-ml conical centrifuge tube, which was then overlaid with the cell suspension, followed by centrifugation for 30 min at 2,000 rpm (724 g) at room temperature without braking. The neutrophils were then collected at the Histopaque-1119 interface. The surface markers of neutrophils (Ly-6G^+^CD11b^+^) were analyzed by flow cytometry.

### Isolation of MSCs From Bone and MSCs Culture

Mesenchymal stem cells (MSCs) from the compact bones of mice were obtained as previously described (Zhu et al., 2010). To deplete hematopoietic cells from the tibiae and femurs, the bone cavities were washed thoroughly at three times using a syringe until the bones become pale. Hold the humeri, tibiae and femurs with forceps and excise the compact bones carefully into chips of approximately 1–3 mm^3^ with scissors. The bone chips were transferred into a 25-cm^2^ plastic culture flask with forceps, then suspend the chips in 3 ml of α-MEM (Hyclone, SH30265.01, United States) containing 10% (vol/vol) FBS (Gibco, 16000-044, United States) in the presence of 1 mg/ml (wt/vol) of collagenase II (Gibco, 17101015, United States). The chips were digested for 1–2 h in a shaking incubator at 37°C with a shaking speed of 200 rpm. The collagenase digestion was stopped when the bone chips become loosely attached to each other. The digestion medium and released cells were aspirated and discarded. Enzyme-treated bone chips were placed in a 10-cm^2^ dish containing 6 ml of α-MEM supplemented with 10% FBS. Each replanting was considered a passage. Passage 3 MSCs were used for all experiments. The surfaces marker of MSCs (Lin^–^CD45^–^CD31^–^CD51^+^Sca-1^+^) were analyzed by Flow cytometry.

### Lin^–^c-kit^+^ (LK^+^) Isolation and Cocultured With MSCs

BM cells were isolated from the tibias, femurs and ilia of 8-week-old *B6.SJL* mice. *wt* LK^+^ cells were sorted with a c-Kit (CD117) Microbead Kit (MACS, 130-091-224, German) and a Lineage Cell Depletion Kit (MACS, 130-090-858, German) according to the manufacturer’s protocol. 1 × 10^6^ LK^+^ cells cultured with 6 × 10^4^ MSCs from *Rheb1*^Δ/Δ^ or *Rheb1^*fl/fl*^* mice for 24 h and counted the number of LK^+^ cells. For the SCF/c-kit blocking experiment, 1 × 10^5^ LK^+^ cells cultured with 6 × 10^4^ MSCs from *Rheb1*^Δ/Δ^ or *Rheb1^*fl/fl*^* mice. SCF inhibitor (MCE, HY-101443, China) was added to the coculture system at 0.5 μM. After 24 h of coculture, counted the number of LK^+^ cells.

### LKS^+^ Isolation and Culture

*Rheb1^*fl/fl*^* or *Rheb*^Δ/Δ^ BM cells were isolated from the tibias, femurs and ilia of 8-week-old mice. LKS^+^ cells were stained with the antibodies indicated above and sorted with a BD FACS Aria III flow cytometer (BD Bioscience, United States). Lin^–^ cells and Lin^–^c-kit^+^ (LK^+^) cells were sorted with a c-Kit (CD117) Microbead Kit (MACS, 130-091-224, Germany) and a Lineage Cell Depletion Kit (MACS, 130-090-858, Germany) according to the manufacturer’s protocol. 2 × 10^5^ *wt* Lin^–^ cells were co-cultured with 2 × 10^7^ *Rheb1^*fl/fl*^* or *Rheb1*^Δ/Δ^ BMCs. The total cells were analyzed for the percentage of CD45.2^+^/CD45.1^+^ cells by flow cytometry.

### MSCs Coculture With Neutrophils

For the neutrophils and MSCs coculture assay, 1 × 10^5^ MSCs were cultured in 24-well plate in a volume of 500 μl α-MEM with 15% FBS. After 24h of culture, the MSCs were cultured with 1 × 10^6^ *Rheb1^*fl/fl*^* or *Rheb1*^Δ/Δ^ neutrophils using cell culture inserts (FALCON, 353095, United States). After 24 h of coculture, the MSCs were harvested, and the relative expression of stem cell factor (SCF) was measured. For the IL-6 neutralization experiment, IL-6 antibody (R&D, MAB406-SP, United States) was added to the coculture system at 10 ng/ml. After 24 h of coculture, MSCs were harvested, and the relative expression of SCF was measured. All cells were incubated at 37°C in a 5% CO_2_ incubator.

### Whole Bone Marrow Transplantation

**FIGURE 1:**
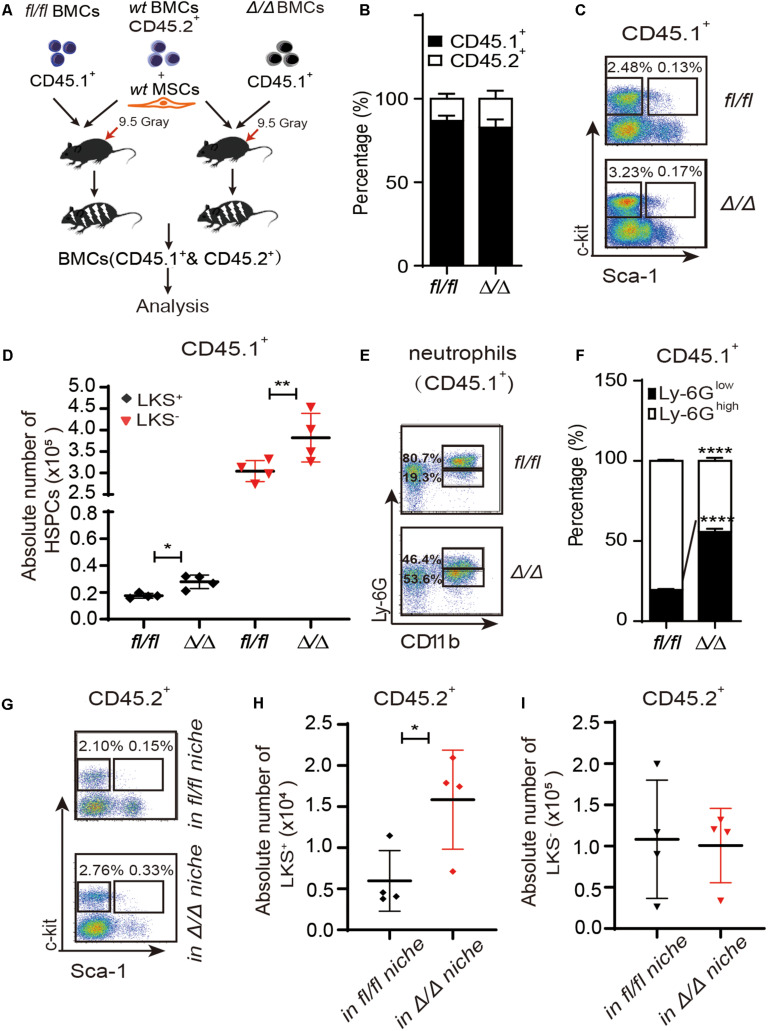
HSPCs are expanded in the *Rheb1*^Δ/Δ^ blood cell-remodeled bone marrow environment. **(A)** *WT* BMCs (CD45.2^+^) together with *wt* MSCs and *Rheb1*^Δ/Δ^ or *Rheb1^*fl/fl*^* BMCs (CD45.1^+^) were transplanted into lethally irradiated recipient *wt* mice. **(B)** The percentages of CD45.1^+^ cells and CD45.2^+^ cells in BM 4 months after transplantation. **(C,D)** The absolute number of CD45.1^+^LKS^+^ cells and CD45.1^+^LKS^–^ cells in BM 4 months after transplantation. **(E,F)** The percentages of CD45.1^+^CD11b^+^Ly-6G^high/low^ neutrophils in BM 4 months after transplantation. **(G–I)** The absolute number of CD45.2^+^LKS^+^ cells and CD45.2^+^LKS^–^ cells in BM 4 months after transplantation. The data are presented as the mean ± SD, *n* = 4. *P < 0.05; **P < 0.01; ***P < 0.001.

For *wt* BMC transplantation, BMCs (CD45.2^+^) were obtained from C57BL/6 mice, and 1 × 10^6^ BMCs were intravenously injected into lethally irradiated 6–8-week-old *Rheb1^*fl/fl*^* or *Rheb1*^Δ/Δ^ recipient mice (CD45.1^+^). For *wt* BMC and *Rheb1*^Δ/Δ^ or *Rheb1^*fl/fl*^* BMC co-transplantation, 5 × 10^5^ *wt* BMCs (CD45.2^+^), 1 × 10^6^ *Rheb1*^Δ/Δ^ or *Rheb1^*fl/fl*^* BMCs (CD45.1^+^) and 7.5 × 10^5^ *wt* MSCs were intravenously injected into lethally irradiated mice (CD45.2^+^) (Figure 1).

### HSPCs Transplantation

200 *wt* LKS^+^ cells (from *B6.SJL* mice, CD45.1^+^) were cocultured with MSCs from *Rheb1*^*fl*/*fl*^ mice or *Rheb1*^Δ/Δ^ mice for 24 h. The cultured LKS^+^ cells (CD45.1^+^) were harvested and intravenously injected into lethally irradiated recipient mice (CD45.2^+^) with 5 × 10^5^ BMCs (CD45.2^+^). The reconstitution of PB cells was analyzed every 4 weeks after transplantation for 4 months, and the reconstitution of BM cells was analyzed at 4 months after transplantation (Figure 4).

**FIGURE 2:**
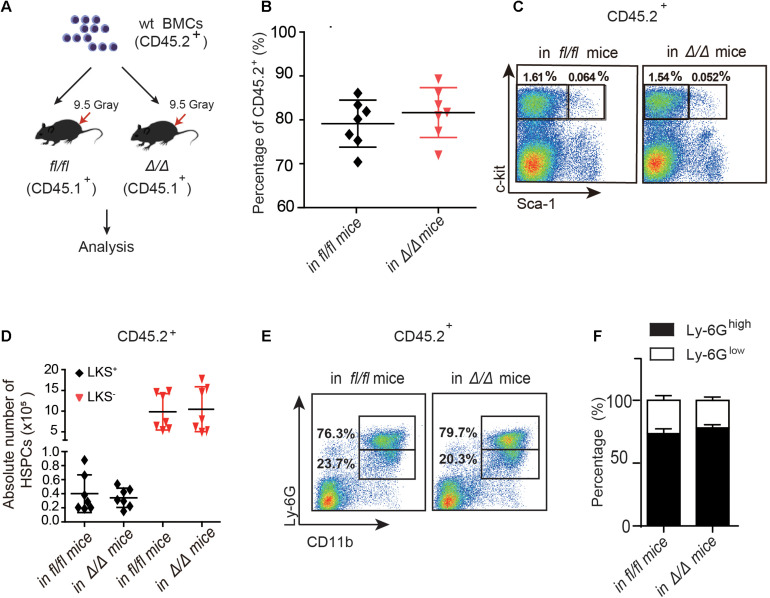
The number of *wt* HSPCs showed no change in *Rheb1*^Δ/Δ^ and *Rheb1^*fl/fl*^* mice at 4 months after transplantation. **(A)** Wild-type (*wt*) whole bone marrow cells (CD45.2^+^) were transplantation into lethally irradiated *Rheb1*^Δ/Δ^ or *Rheb1^*fl/fl*^* mice. **(B)** The percentage of donor-derived cells (CD45.2^+^) in PB 4 months after transplantation. **(C,D)** The absolute number of donor-derived LKS^+^ and LKS^+^ cells (CD45.2^+^) in BM 4 months after transplantation. **(E,F)** The percentage of donor-derived CD11b^+^Ly-6G^high/low^ cells (CD45.2^+^) in BM 4 months after transplantation. The data are presented as the mean ± SD, *n* = 7.

**FIGURE 3:**
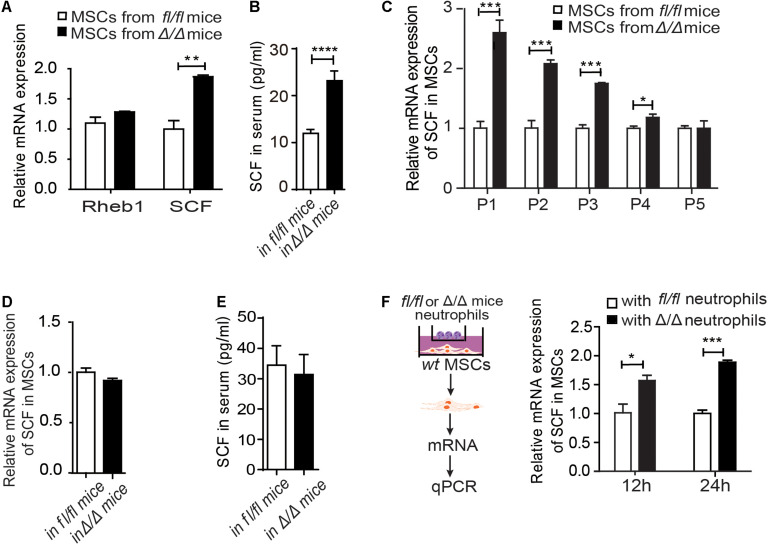
*Rheb1*-deficient neutrophils stimulate MSCs to secrete more SCF. **(A)** The relative mRNA expression of Rheb1 and SCF in primary MSCs from *Rheb1^*fl/fl*^* and *Rheb1*^Δ/Δ^ mice (*n* = 3). **(B)** The serum levels of SCF in *Rheb1^*fl/fl*^* and *Rheb1*^Δ/Δ^ mice (*n* = 4). **(C)** The relative mRNA expression of SCF in passage 1 (P1) to P5 cultured MSCs from *Rheb1^*fl/fl*^* and *Rheb1*^Δ/Δ^ mice (*n* = 3). **(D)** The relative mRNA expression of SCF in MSCs from *Rheb1^*fl/fl*^* and *Rheb1*^Δ/Δ^ mice 4 months after *wt* BMC transplantation (*n* = 3). **(E)** The serum level of SCF in *Rheb1^*fl/fl*^* or *Rheb1*^Δ/Δ^ mice 4 months after *wt* BMC transplantation (*n* = 3). **(F)** The relative mRNA expression of SCF in *wt* MSCs cocultured with *Rheb1^*fl/fl*^* or *Rheb1*^Δ/Δ^ neutrophils after 12 h and 24 h (*n* = 3). The data are presented as the mean ± SD. *P < 0.05; **P < 0.01; ***P < 0.001; ****P < 0.0001.

**FIGURE 4:**
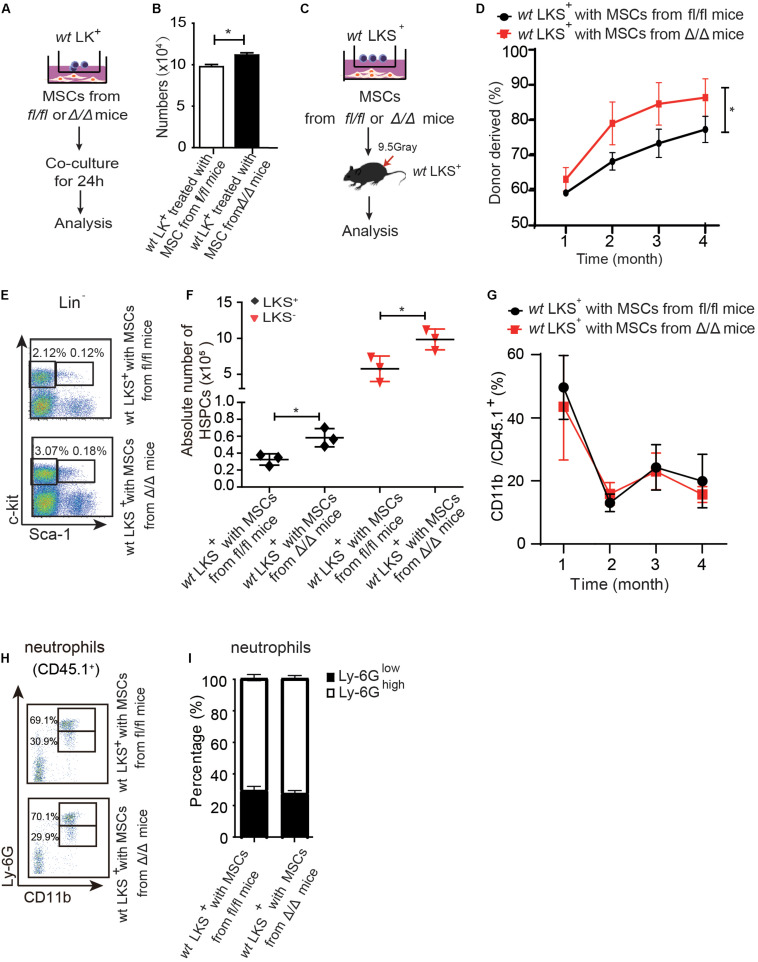
*Rheb1*-deficient neutrophil trained-MSCs promote *wt* HSPCs expansion. **(A,B)** The number of LK^+^ cells after coculturing with MSCs from *Rheb1^*fl/fl*^* and *Rheb1*^Δ/Δ^ mice for 24 h. **(C)** *wt* LKS^+^ (CD45.1^+^) were cultured with MSCs from *Rheb1*^Δ/Δ^ or *Rheb1^*fl/fl*^* mice for 24 h, and the cultured LKS^+^ (CD45.1^+^) cells and newly isolated *wt* WBMCs (CD45.2^+^) were transplanted into lethally irradiated recipient mice by intravenously injection. **(D)** The percentage of donor-derived LKS^+^ cells (CD45.1^+^) in PB 4 months after transplantation. The data are presented as the mean ± SD, *n* = 5. **(E,F)** The absolute number of donor-derived LKS^+^ cells (CD45.1^+^) in BM (*n* = 3). **(G)** The percentage of donor-derived CD11b^+^ cells (CD45.1^+^) in PB. The data are presented as the mean ± SD, *n* = 5. **(H,I)** The percentage of donor-derived CD11b^+^Ly-6G^ high/low^ neutrophils (CD45.1^+^) in BM 4 months after transplantation. The data are presented as the mean ± SD. *P < 0.05.

### Isolation of MSCs and MNCs From CML Patients

CML patients’ BM cells were obtained from the Blood Bank of the State Key Laboratory of Experimental Hematology, Institute of Hematology and Blood Diseases Hospital, CAMS/PUMC. Specimen acquisition was approved by the Ethics Committee of Blood Diseases Hospital, Chinese Academy of Medical Sciences. All donors signed informed consent forms. One-fifth the volume of hydroxyethyl starch was added to the patient samples, and let stand for 1 h at room temperature to allow the red blood cells to fully sediment. Gently suck the supernatant and divide it into two according to the volume of 3:1. A large volume of supernatant fluid was selected to culture MSC in containing 6 ml of α-MEM supplemented with 10% (vol/vol) FBS. The other was separated using a Ficoll (GE, United States) gradient to generated mononuclear cells (MNCs).

### Quantitative Real-Time PCR

RNA was extracted using the RNeasy Mini Kit (QIAGEN, 74106, Germany) according to the manufacturer’s protocol. cDNA synthesis was performed using a cDNA reverse transcription kit (Takara, RR047A, Japan) according to the manufacturer’s protocol. Quantitative PCR assays were performed in 96-well Micro Amp Fast Optical Reaction Plates (Applied Biosystems, 4344904, United States) using SYBR Green Mix (Roche, 04913914001, Switzerland). The signal was detected using the Step-One Plus Real-Time PCR System (QuantStudio5). GAPDH was used as an endogenous control for gene expression assays.

### ELISA

500 μl PB was obtained from the retro-orbital bleeding of mice and clotted for 1 h at room temperature, then centrifuged at 5,915 rpm (3,000 g) for 10 min, and the serum was collected for determination. Then ELISA was performed using the Mouse SCF ELISA Kit (Quantikine, MCK00, United States) and the Mouse IL-6 ELISA Kit (NRC, TAE-385, Canada) according to the manufacturer’s protocols. A total of 5 × 10^6^ *Rheb1^*fl/fl*^* or *Rheb1*^Δ/Δ^ neutrophils in 200 μl of PBS were frozen and thawed three times and centrifuged at 5,915 rpm (3,000 g) for 10 min, and the liquid supernatants were collected for IL-6 determination. The cell culture medium was concentrated with an ultra-gentrification device (Merck, UFC900308, Germany). The ELISA tests were read on a Synergy^*H*4^ Hybrid Reader at 450 nm.

### Statistical Analysis

GraphPad Prism 6.0 was used for statistical analyses. Every experiment was compared as two groups. The data are presented as the mean ± standard deviation (SD). The unpaired two-tailed Student’s *t*-test was used to compute the *P*-values. *P* < *0.05* was considered significant. Significant differences are indicated with asterisks (*^∗^P* < *0.05; ^∗∗^P* < *0.01; ^∗∗∗^P* < *0.001*).

## Results

### HSPCs Are Expanded in the *Rheb1*^Δ/Δ^ Blood Cell-Remodeled Bone Marrow Environment

We have shown that *Vav1-Cre;Rheb1^*fl/fl*^* mice present HSCs and immature myeloid cells expansion in BM, which resemble the phenotype of MPN patients (Wang et al., 2018). To investigate whether *Rheb1*-deficient cells regulate the proliferation of HSPCs, we established a chimeric mouse model with *wt* HSPCs and a *Rheb1*^Δ/Δ^ blood cell-remodeled bone marrow environment. We isolated MSCs from bone of *wt* mice (*wt* MSCs, Supplementary Figure 1B), and transplanted 5 × 10^5^ *wt* BMCs (CD45.2^+^) together with 7.5 × 10^5^ *wt* MSCs and 1 × 10^6^ *Rheb1*^Δ/Δ^ or *Rheb1^*fl/fl*^* BMCs (CD45.1^+^) into lethally irradiated recipient *wt* mice (CD45.2) (Figure 1A). The percentage of *Rheb1*^Δ/Δ^ and *Rheb1^*fl/fl*^* BMC-derived cells (CD45.1^+^) in PB was approximately 80%, 4 months after the transplantation, and the percentage of *wt* BMC-derived cells (CD45.2^+^) in PB was close to 20% in mice after the transplantation (Figure 1B and Supplementary Figure 1C). The absolute number of *Rheb1*^Δ/Δ^ -derived LKS^+^ and LKS^–^ cells (CD45.1^+^) were significantly increased when compared with those of *Rheb1^*fl/fl*^*-derived LKS^+^ and LKS^–^ cells (CD45.1^+^), respectively (Figures 1C,D). *Rheb1*^Δ/Δ^ BMC-derived myeloid cells were also increased in the PB of mice (Supplementary Figure 1D). Moreover, the percentage of *Rheb1*^Δ/Δ^ Ly-6G^low^CD11b^+^ immature neutrophils (CD45.1^+^) was increased in mice when compared with that of *Rheb1^*fl/fl*^* Ly-6G^low^CD11b^+^ immature neutrophils (Figures 1E,F). These phenotypes were similar with *Rheb1*^Δ/Δ^ mouse (Wang et al., 2018). Then we analyzed the absolute number of *wt* HSPCs (CD45.2^+^) in the *Rheb1*^Δ/Δ^ blood cell-remodeled bone marrow environment in the chimeric mouse model, and found that the absolute number of *wt* LKS^+^ cells (CD45.2^+^) transplanted with *Rheb1*^Δ/Δ^ BMCs were higher than those of the cells transplanted with *Rheb1^*fl/fl*^* BMCs (Figures 1G–I). The percentage of *wt* BMC-derived myeloid cells (CD45.2^+^) was increased in PB, while the ratio of *wt* BMC-derived Ly-6G^high^CD11b^+^ and Ly-6G^low^CD11b^+^ neutrophils (CD45.2^+^) was not changed in the BM of mice co-transplanted with *Rheb1*^Δ/Δ^ BMCs (Supplementary Figures 1E,F). It indicated that *Rheb1*^Δ/Δ^ blood cell remodeled bone marrow environment and promoted HSPC proliferation. To explore whether *Rheb1*^Δ/Δ^ BM cells directly promoted phenotypic HSPCs over proliferation, we cocultured *wt* Lin^–^ cells (CD45.2^+^) with BMCs from *Rheb1^*fl/fl*^* and *Rheb1*^Δ/Δ^ mice (CD45.1^+^) (Supplementary Figure 2A). Interestingly, the number of *wt* Lin^–^ cells cocultured with BMCs from *Rheb1*^Δ/Δ^ mice was similar with that in the control group at 24 h (Supplementary Figure 2B). These data suggested that *Rheb1*^Δ/Δ^ BM cells indirectly promoted HSPCs over-proliferation.

### The Proliferation of *wt* HSPCs Was Normal in *Rheb1*^Δ/Δ^ Recipient Mice After Transplantation

To investigate whether the niche cells affect the expansion of HSPCs in *Rheb1*^Δ/Δ^ mice, we transplanted *wt* whole bone marrow cells (CD45.2^+^) into lethally irradiated *Rheb1*^Δ/Δ^ or *Rheb1^*fl/fl*^* recipient mice (CD45.1^+^) and analyzed the donor-derived HSPCs (CD45.2^+^) at 4 months after transplantation (Figure 2A). The rate of donor chimerism was approximately 80% in both *Rheb1*^Δ/Δ^ and control mice (Figure 2B). The absolute number of donor-derived LKS^+^ cells and LKS^–^ cells (CD45.2^+^) in *Rheb1*^Δ/Δ^ mice were similar when compared with those in the control mice (Figures 2C,D). Since *Rheb1* deletion caused increased number and immaturity of neutrophils in steady condition (Wang et al., 2018), we also analyzed neutrophils by flow cytometry (FACS) with CD11b and Ly-6G antibodies, that have been used as neutrophil subpopulation markers for the identification of myelocytes or promyelocytes, as well as immature or mature neutrophils. The CD11b^+^Ly-6G^+^ subpopulation of donor-derived cells (*wt*) in *Rheb1*^Δ/Δ^ mice was similar to that in the control mice (Figure 2E,F). These data indicated that niche cells in *Rheb1*^Δ/Δ^ mice contributed little to HSCs proliferation *in vivo*, while *Rheb1*^Δ/Δ^ BM cells cooperated with niche cells to promote HSCs proliferation.

### *Rheb1*-Deficient Neutrophils Stimulate MSCs to Secrete More SCF

To investigate whether *Rheb1*-deficient BM cells interacted with MSCs and stimulated HSCs expansion, we evaluated the expression of Rheb1 and SCF in MSCs from *Rheb1*^Δ/Δ^ mice. We found that the mRNA expression of SCF was higher in primary MSCs from *Rheb1*^Δ/Δ^ mice than that from *Rheb1^*fl/fl*^* mice, while Rheb1 expression was equal in these two cell types (Figure 3A). The SCF level in serum was also increased in *Rheb1*^Δ/Δ^ mice when compared with that in *Rheb1^*fl/fl*^* mice (Figure 3B), while the expression of EPO, G-CSF, TPO IL-3 and IL-6 showed no difference between two groups (Supplementary Figure 3A). Furthermore, we cultured MSCs derived from *Rheb1^*fl/fl*^* and *Rheb1*^Δ/Δ^ mice *in vitro* and analyzed the expression of SCF in MSCs after serial passages. We found that in comparison with the MSCs from *Rheb1^*fl/fl*^* mice, the expression of SCF remained at a higher level in MSCs from *Rheb1*^Δ/Δ^ mice at passage 3 and then decreased to about the same level as in MSCs from that of *Rheb1^*fl/fl*^* mice at passage 5 (Figure 3C). Interestingly, when *wt* BMCs were transplanted into lethally irradiated *Rheb1^*fl/fl*^* and *Rheb1*^Δ/Δ^ mice (Figure 2A), SCF mRNA expression in MSCs and SCF serum levels were restored to normal levels (Figures 3D–E). These results demonstrated that the higher expression of SCF in MSCs from *Rheb1*^Δ/Δ^ mice was caused by extrinsic factors from the bone marrow cells of *Rheb1*^Δ/Δ^ mice and was reversible under native conditions.

To evaluate the specific role(s) of *Rheb1*^Δ/Δ^ BMCs in influencing MSCs, we cocultured *wt* MSCs with *Rheb1*^Δ/Δ^ or *Rheb1^*fl/f*^* T cells, B cells and myeloid cells for 12 h and then analyzed the expression of SCF in MSCs under each condition. Interestingly, the expression of SCF was higher in MSCs in the presence of *Rheb1*^Δ/Δ^ myeloid cells than in the presence of *Rheb1*^Δ/Δ^ T cells or B cells (Supplementary Figure 3B). In our previous study, we showed that the differentiation of neutrophils was abnormal in the BM of *Rheb1*^Δ/Δ^ mice. Hence, we isolated neutrophils (Supplementary Figure 3C) from bone marrow cells and cocultured *wt* MSCs with *Rheb1^*fl/fl*^* and *Rheb1*^Δ/Δ^ neutrophils for 24 h. We found that the expression of SCF was significantly increased in MSCs in the presence of *Rheb1*^Δ/Δ^ neutrophils after cultured for 24 h when compared with the control (Figure 3F). Taken together, our results suggested that *Rheb1*^Δ/Δ^ neutrophils stimulated MSCs to produce more SCF.

### *Rheb1*-Deficient Neutrophil-Trained-MSCs Promote *wt* HSPCs Expansion

We then cocultured 1 × 10^6^ LK^+^ cells with 6 × 10^4^ MSCs from *Rheb1*^Δ/Δ^ or *Rheb1^*fl/fl*^* mice for 24 h and found that the number of LK^+^ cells cocultured with MSCs from *Rheb1*^Δ/Δ^ mice was more than the number of LK^+^ cells cocultured with MSCs from *Rheb1^*fl/fl*^* mice (Figures 4A,B). Next, we blocked SCF/c-kit signaling by adding a SCF inhibitor in the culture medium. We found that the number of LK^+^ cells was equally decreased in coculturing with MSCs from *Rheb1*^Δ/Δ^ or *Rheb1^*fl/fl*^* mice in medium with SCF inhibitor (Supplementary Figures 3D,E). These results indicated that *Rheb1*-deficient neutrophil-trained MSCs could promote *wt* HSPCs expansion through SCF signaling pathway.

We further cocultured 200 *wt* LKS^+^ (CD45.1^+^) with 2 × 10^4^ MSCs from *Rheb1*^Δ/Δ^ or *Rheb1^*fl/fl*^* mice for 24 h, then transplanted the cultured LKS^+^ (CD45.1^+^) cells with 5 × 10^5^ newly isolated *wt* WBMCs (CD45.2^+^) into lethally irradiated recipient mice by intravenously injection respectively (Figure 4C). We found that the percentage of chimerism was higher in mice transplanted with LKS^+^ cells cocultured with MSCs from *Rheb1*^Δ/Δ^ mice when compared with those from *Rheb1^*fl/fl*^* mice (Figure 4D). Accordingly, the absolute number of LKS^+^ (CD45.1^+^) cells treated with MSCs from *Rheb1*^Δ/Δ^ mice was significantly increased in comparison with the controls at 4 months after transplantation (Figures 4E,F). However, the percentage of CD11b^+^ (CD45.1^+^) myeloid cells was similar in the PB of the two groups (Figure 4G). The ratio of Ly-6G^high^CD11b^+^ and Ly-6G^low^CD11b^+^ neutrophils was also not changed between the two groups (Figures 4H,I). These data indicated that HSPCs cocultured with MSCs from *Rheb1*^Δ/Δ^ mice exhibited a higher expansion capacity while their ability to differentiate into myeloid cells was not changed. MSCs educated by *Rheb1*-deficient neutrophils induced LKS^+^ over proliferation but not myeloid differentiation.

### *Rheb1*-Deficient Neutrophils Stimulate MSCs to Produce More SCF by Decreasing IL-6 Expression

**FIGURE 5:**
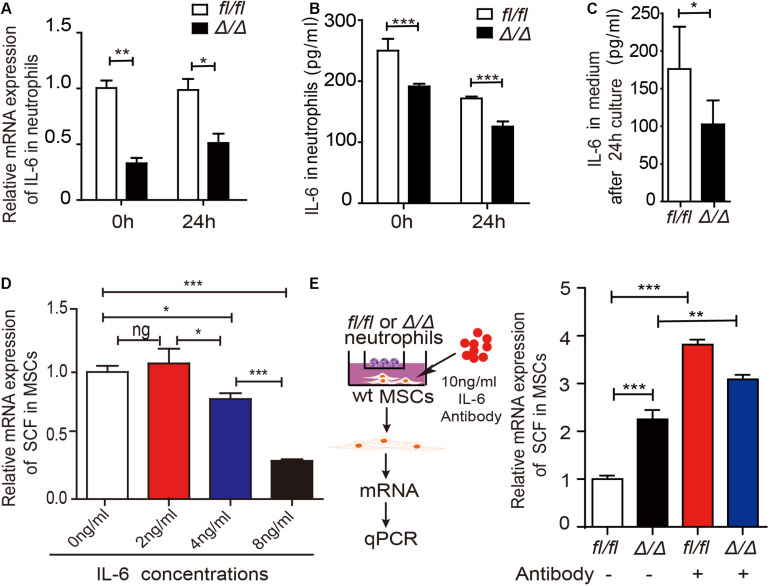
*Rheb1*-deficient neutrophils stimulate MSCs to produce more SCF by decreasing IL-6 expression. **(A)** The relative mRNA expression of IL-6 in *Rheb1^*fl/fl*^* or *Rheb1*^Δ/Δ^ neutrophils when separated from BM (0 h) or after 24 h of coculture with MSCs (*n* = 3). **(B)** The protein levels of IL-6 in the cell lysates of *Rheb1^*fl/fl*^* and *Rheb1*^Δ/Δ^ neutrophils when separated from BM (0 h) or after 24 h of coculture with MSCs (*n* = 3). **(C)** The IL-6 levels in the media of *Rheb1^*fl/fl*^* or *Rheb1*^Δ/Δ^ neutrophils cocultured with MSCs (*n* = 4). **(D)** The relative mRNA expression of SCF in *wt* MSCs after exposure to different concentrations IL-6 for 24 h (*n* = 3). **(E)** The relative mRNA expression of SCF in *wt* MSCs cocultured with *Rheb1^*fl/fl*^* or *Rheb1*^Δ/Δ^ neutrophils after being exposed to an IL-6 antibody at 10 ng/ml for 24 h (*n* = 3). *P < 0.05; **P < 0.01; ***P < 0.001.

To investigate the underlying mechanisms by which *Rheb1*-deficient neutrophils stimulate MSCs to increase SCF production, we measured the mRNA expression of several potential interleukin and chemokine candidates secreted by neutrophils (Supplementary Figure 3F). We found that the mRNA and protein expression levels of IL-6 were decreased in *Rheb1*^Δ/Δ^ neutrophils when compared with those from the control (Figures 5A,B, 0 h). We confirmed that IL-6 is mainly expressed in myeloid cells (Supplementary Figure 3G), and found that IL-6 mRNA and protein expression levels in *Rheb1*^Δ/Δ^ neutrophils remained lower after cultured with *wt* MSCs for 24 h *in vitro* (Figures 5A,B, 24 h). In addition, the level of IL-6 in the *Rheb1*^Δ/Δ^ neutrophil coculture medium was also lower than that in the control (Figure 5C). These data indicated that *Rheb1*^Δ/Δ^ neutrophils expressed and secreted less IL-6 than *Rheb1^*fl/fl*^* neutrophils. Since MSCs are immunomodulatory cells and secrete a variety of cytokines, including IL-6, we measured the mRNA expression of IL-6 in MSCs and found that its expression in MSCs from *Rheb1*^Δ/Δ^ mice was similar to that in MSCs isolated from *Rheb1^*fl/fl*^* mice (Supplementary Figure 3H). To determine the role of IL-6 in MSCs, we cultured *wt* MSCs with different concentrations of IL-6 and analyzed the relative SCF mRNA expression in MSCs at 24 h. We found that higher IL-6 treatment suppressed SCF mRNA expression in MSCs (Figure 5D). We then blocked IL-6 signaling by adding an IL-6 neutralizing antibody in the media in which *Rheb1*^Δ/Δ^ or *Rheb1^*fl/fl*^* neutrophils were cocultured with *wt* MSCs, and measured the expression of SCF mRNA in MSCs (Figure 5E, left panel). We found that the mRNA expression of SCF was significantly increased in MSCs after treatment with the IL-6-neutralizing antibody in coculture with either *Rheb1^*fl/fl*^* or *Rheb1*^Δ/Δ^ neutrophils (Figure 5E, right panel). These data suggested that the lower expression of IL-6 in *Rheb1*^Δ/Δ^ neutrophils stimulated the expression of SCF in MSCs.

### IL-6 Regulates SCF Expression in Human MSCs

**FIGURE 6:**
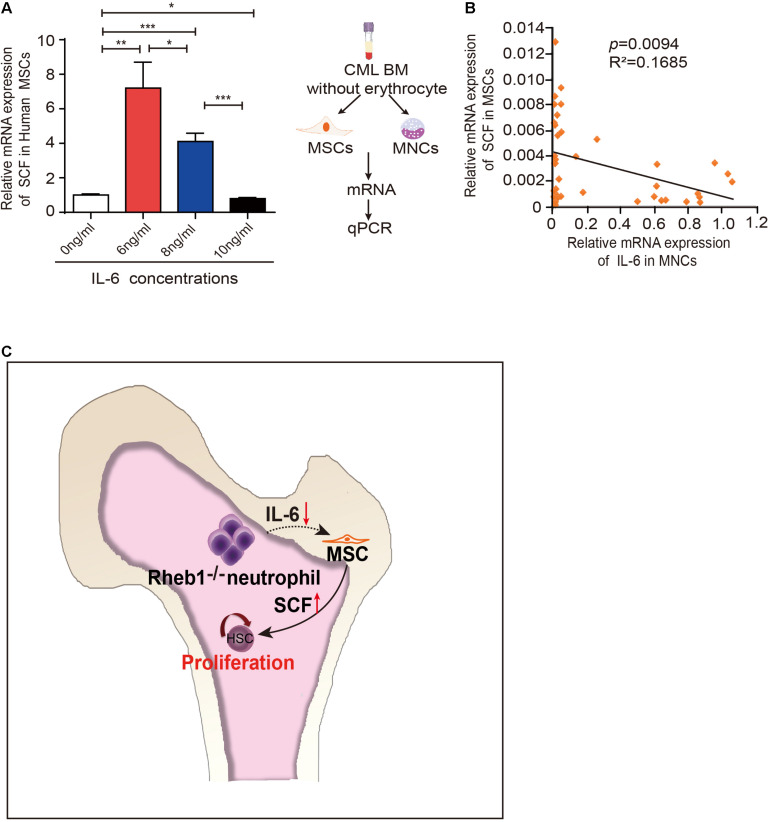
IL-6 regulates SCF expression in human MSCs. **(A)** The relative mRNA expression of SCF in H-MSCs after exposure to different concentrations IL-6 for 24 h (*n* = 3). The data are presented as the mean ± SD. **(B)** Correlation analysis for SCF and IL-6 mRNA expression in CML patients (*n* = 39) (*p* = 0.0094). R: Pearson correlation coefficients; R^2^: indicates “the goodness of fit.” Statistical significance was calculated by Pearson correlation coefficients. **(C)** Model depicting hematopoietic regulation in the absence of *Rheb1*. *P < 0.05; **P < 0.01; ***P < 0.001.

It has been reported that IL-6 was higher in CML patients (Panteli et al., 2005) and higher level of IL-6 is associated with poor prognosis in CML patients (Nievergall et al., 2016). To evaluate the relationship of IL-6 and SCF expression level in human cells, we first investigated whether IL-6 regulates SCF expression in human MSCs. We cultured MSCs from human umbilical cord blood with different concentrations of IL-6 and analyzed the relative SCF mRNA expression in MSCs at 24 h. The result showed that higher IL-6 treatment suppressed SCF mRNA expression in human MSCs (Figure 6A). Next we isolated MNCs and MSCs from the BM of 39 CML patients, and measured the expression of IL-6 in mononuclear cells and the expression of SCF in MSCs in patients (Figure 6B, left panel). We found a negative correlation of SCF and IL-6 by analyzing the relationship of IL-6 and SCF expression in CML patients (*n* = 39) (Figure 6B, right panel). The data indicated that higher expression of IL-6 in myeloid cells is associated with the lower expression of SCF in MSCs in CML patients.

## Discussion

We previously found that the HSPCs were over-expanded in *Vav1-Cre;Rheb1^*fl/fl*^* mice (Wang et al., 2018). Here, using *in vivo* and *in vitro* models, we found that loss of *Rheb1* impaired neutrophils’ ability to secrete IL-6, which stimulated MSCs to secrete more SCF and in turn stimulated HSPCs to proliferate (Figure 6C).

The microenvironment in BM comprises multipotent stromal cells (MSCs), osteoblasts, endothelial cells, mature blood cells and the cytokines that they produced (Gao et al., 2018). We previously showed that the differentiation and maturation of neutrophils in BM were abnormal in *Rheb1*^Δ/Δ^ HSCs, leading aged *Rheb1*^Δ/Δ^ mice to show MPN-like symptoms (Wang et al., 2018). Neutrophils and their progenitors have been shown to secrete a variety of cytokines to influence the development and proliferation of HSCs either directly or indirectly. For example, apoptotic neutrophils can stimulate macrophages to secrete G-CSF and regulate HSCs indirectly in zebrafish (Hall et al., 2016). Gr-1^+^ neutrophils in mouse bone marrow produce ROS during acute infection, which contributes to the proliferation of HSPCs via a paracrine mechanism (Kwak et al., 2015). Senescent neutrophils are markedly increased in aged mice and promote an increase in platelet-biased HSCs via IL-1β (Frisch et al., 2019). Our results demonstrated that *Rheb1*^Δ/Δ^ neutrophils remodeled the BM niche, and induced HSPCs to undergo overexpansion in BM (Figure 3) probably via a feedback in MSC through the proinflammatory factor IL-6 (Figures 4, 5). This partially supported that the abnormal myeloid cells could regulate the proliferation of HSCs in MPN patients (Schepers et al., 2013).

IL-6 was higher in CML patients and was elevated during the transformation phase of CML (Panteli et al., 2005), while blocking IL-6 signaling could delay CML development in mouse model (Reynaud et al., 2011). More importantly, higher level of IL-6 is shown to be associated with poor prognosis in CML patients (Nievergall et al., 2016). We found there is a negative correlation of SCF with IL-6 in CML patients (Figure 6B), indicating that higher IL-6 could inhibit MSCs to secrete SCF and impair normal HSPC proliferation, thus promote CML progression. It suggested that blocking IL-6 signaling might be benefit for the restoration of HSC proliferation in CML patients.

*Rheb1* has been implicated in many metabolic processes via the canonical TSC/Rheb/mTOR signaling pathway and/or non-canonical signaling pathways. We previously reported that *Rheb1* deficiency inhibited neutrophil maturation through the mTOR signaling pathway (Wang et al., 2018). We found mTORC1 inhibitor Rapamycin could reduce p-S6 level and IL-6 RNA expression level in neutrophils (data not shown), which was consistent with blocking Rheb1-mTORC1 signaling pathway decreased IL-6 expression in IL-33 stimulated type 2 innate lymphoid cells (ILC2) (Petrova et al., 2020). Since IL-6 is a pleiotropic cytokine and a strong activator of Mammalian Target of Rapamycin (mTOR) (Pinno et al., 2016), it is possible that *Rheb1* regulates IL-6 expression through a negative feedback loop affecting mTORC1. Several studies revealed that the inhibition of B-Raf and Dyneinor activation of the Notch signaling pathway by *Rheb1* is independent of the mTORC1 pathway (Neuman and Henske, 2011), and increased Notch signaling increases IL-6 expression, leading to the activation of IL-6/JAK/STAT signaling (Jin et al., 2013). The specific role and mechanism of *Rheb1* in the IL-6 signaling pathway in neutrophils need to be further investigated.

## Data Availability Statement

The original contributions presented in the study are included in the article/Supplementary Material, further inquiries can be directed to the corresponding author/s.

## Ethics Statement

The studies involving human participants were reviewed and approved by the Ethics Committee of Blood Diseases Hospital, Chinese Academy of Medical Sciences. The patients/participants provided their written informed consent to participate in this study. The animal study was reviewed and approved by Institutional Animal Care and Use Committee (IACUC), Institute of Hematology and Blood Diseases Hospital (CAMS/PUMC).

## Author Contributions

JG, SH, and XW performed the experiments, analyzed the data, and wrote the manuscript. SY, YW, YG, and XS helped with the *in vivo* experiments and data collection. WW, YC, YZ, XF, HL, TC, and JS contributed to the data analyses and manuscript discussion. XW and WY conceived and directed the research project and revised the manuscript. All authors contributed to the article and approved the submitted version.

## Conflict of Interest

The authors declare that the research was conducted in the absence of any commercial or financial relationships that could be construed as a potential conflict of interest.

## Abbreviations

BMCs: whole bone marrow cells
BM: bone marrow
MNCs: mononuclear cells
PB: peripheral blood
HSC: hematopoietic stem cell
HPC: hematopoietic progenitor cell
LKS^±^: Lin^–^c-kit^+^Sca-1^±^
LK^+^: Lin^–^c-kit^+^
MSC: mesenchymal stem cell
OBC: osteoblast cell
*wt*: wild-type
SCF: stem cell factor
CML: chronic myeloid leukemia
PBS: Phosphate Buffer Solution
FBS: fetal bovine serum
HBSS: Hanks’ Balanced Salt Solution.

## References

[B1] Arranz L. Sanchez-Aguilera A. Martin-Perez D. Isern J. Langa X. Tzankov A. (2014). Neuropathy of haematopoietic stem cell niche is essential for myeloproliferative neoplasms. *Nature* 512 78–81. 10.1038/nature13383 25043017

[B2] Aspuria P. J. Tamanoi F. (2004). The Rheb family of GTP-binding proteins. *Cell. Signal.* 16 1105. 10.1016/j.cellsig.2004.03.019 15240005

[B3] Chow A. Lucas D. Hidalgo A. Mendez-Ferrer S. Hashimoto D. Scheiermann C. (2011). Bone marrow CD169+ macrophages promote the retention of hematopoietic stem and progenitor cells in the mesenchymal stem cell niche. *J. Exp. Med.* 208 261–271. 10.1084/jem.20101688 21282381 PMC3039855

[B4] Feng C. G. Weksberg D. C. Taylor G. A. Sher A. Goodell M. A. (2008). The p47 GTPase Lrg-47 (Irgm1) links host defense and hematopoietic stem cell proliferation. *Cell Stem Cell* 2 83–89. 10.1016/j.stem.2007.10.007 18371424 PMC2278017

[B5] Frenette P. S. Pinho S. Lucas D. Scheiermann C. (2013). Mesenchymal stem cell: keystone of the hematopoietic stem cell niche and a stepping-stone for regenerative medicine. *Annu. Rev. Immunol.* 31 285–316. 10.1146/annurev-immunol-032712-095919 23298209

[B6] Frisch B. J. Hoffman C. M. Latchney S. E. LaMere M. W. Myers J. Ashton J. (2019). Aged marrow macrophages expand platelet-biased hematopoietic stem cells via Interleukin1. *JCI Insight* 5:e124213. 10.1172/jci.insight.124213 PMC6542605 30998506

[B7] Gao X. Xu C. Asada N. Frenette P. S. (2018). The hematopoietic stem cell niche: from embryo to adult. *Development* 145:dev139691. 10.1242/dev.139691 29358215 PMC5825844

[B8] Gao Y. Gao J. Li M. Zheng Y. Wang Y. Zhang H. (2016). Rheb1 promotes tumor progression through mTORC1 in MLL-AF9-initiated murine acute myeloid leukemia. *J. Hematol. Oncol.* 9:36. 27071307 10.1186/s13045-016-0264-3 PMC4830070

[B9] Greenbaum A. Hsu Y. M. Day R. B. Schuettpelz L. G. Christopher M. J. Borgerding J. N. (2013). CXCL12 in early mesenchymal progenitors is required for haematopoietic stem-cell maintenance. *Nature* 495 227–230. 10.1038/nature11926 23434756 PMC3600148

[B10] Hall C. Crosier P. Crosier K. (2016). Inflammatory cytokines provide both infection-responsive and developmental signals for blood development: lessons from the zebrafish. *Mol. Immunol.* 69 113–122. 10.1016/j.molimm.2015.10.020 26563946

[B11] Jin S. Mutvei A. P. Chivukula I. V. Andersson E. R. Ramskold D. Sandberg R. (2013). Non-canonical Notch signaling activates IL-6/JAK/STAT signaling in breast tumor cells and is controlled by p53 and IKKalpha/IKKbeta. *Oncogene* 32 4892–4902. 10.1038/onc.2012.517 23178494 PMC3795477

[B12] Kwak H. J. Liu P. Bajrami B. Xu Y. Park S. Y. Nombela-Arrieta C. (2015). Myeloid cell-derived reactive oxygen species externally regulate the proliferation of myeloid progenitors in emergency granulopoiesis. *Immunity* 42 159–171. 10.1016/j.immuni.2014.12.017 25579427 PMC4303526

[B13] Meng Z. Perry J. M. Heather M. Aparna V. Pengxu Q. He X. C. (2014). Megakaryocytes maintain homeostatic quiescence and promote post-injury regeneration of hematopoietic stem cells. *Nat. Med.* 20 1321–1326. 10.1038/nm.3706 25326798

[B14] Neuman N. A. Henske E. P. (2011). Non-canonical functions of the tuberous sclerosis complex-Rheb signalling axis. *EMBO Mol. Med.* 3 189–200. 10.1002/emmm.201100131 21412983 PMC3377068

[B15] Nievergall E. Reynolds J. Kok C. H. Watkins D. B. Biondo M. Busfield S. J. (2016). TGF-α and IL-6 plasma levels selectively identify CML patients who fail to achieve an early molecular response or progress in the first year of therapy. *Leukemia* 30 1263–1272. 10.1038/leu.2016.34 26898188

[B16] Panteli K. E. Hatzimichael E. C. Bouranta P. K. Katsaraki A. Seferiadis K. Stebbing J. (2005). Serum interleukin (IL)-1, IL-2, sIL-2Ra, IL-6 and thrombopoietin levels in patients with chronic myeloproliferative diseases. *Br. J. Haematol.* 130 709–715. 10.1111/j.1365-2141.2005.05674.x 16115126

[B17] Petrova T. Pesic J. Pardali K. Gaestel M. Arthur J. S. C. (2020). p38 MAPK signalling regulates cytokine production in IL-33 stimulated Type 2 Innate Lymphoid cells. *Sci. Rep.* 10:3479. 32103032 10.1038/s41598-020-60089-0 PMC7044202

[B18] Pinno J. Bongartz H. Klepsch O. Wundrack N. Poli V. Schaper F. (2016). Interleukin-6 influences stress-signalling by reducing the expression of the mTOR-inhibitor REDD1 in a STAT3-dependent manner. *Cell. Signal.* 28 907–916. 10.1016/j.cellsig.2016.04.004 27094713

[B19] Raaijmakers M. H. (2012). Myelodysplastic syndromes: revisiting the role of the bone marrow microenvironment in disease pathogenesis. *Int. J. Hematol.* 95 17–25. 10.1007/s12185-011-1001-x 22218882

[B20] Rathinam C. Flavell R. A. (2008). The hematopoiesis paradigm: Clarity or ambiguity? *Blood* 112 3534–3535. 10.1182/blood-2008-07-167759 18948582 PMC2572785

[B21] Reynaud D. Pietras E. Barry-Holson K. Mir A. Binnewies M. Jeanne M. (2011). IL-6 controls leukemic multipotent progenitor cell fate and contributes to chronic myelogenous leukemia development. *Cancer Cell* 20 661–673. 10.1016/j.ccr.2011.10.012 22094259 PMC3220886

[B22] Schepers K. Pietras E. M. Reynaud D. Flach J. Binnewies M. Garg T. (2013). Myeloproliferative neoplasia remodels the endosteal bone marrow niche into a self-reinforcing leukemic niche. *Cell Stem Cell* 13 285–299. 10.1016/j.stem.2013.06.009 23850243 PMC3769504

[B23] Swamydas M. Luo Y. Dorf M. E. Lionakis M. S. (2015). Isolation of mouse neutrophils. *Curr. Protoc. Immunol.* 110 3.20.1–3.20.15. 26237011 10.1002/0471142735.im0320s110 PMC4574512

[B24] Wang X. Chu Y. Wang W. Yuan W. (2016a). mTORC signaling in hematopoiesis. *Int. J. Hematol.* 103 510–518. 26791377 10.1007/s12185-016-1944-z

[B25] Wang X. Gao Y. Gao J. Li M. Zhou M. Wang J. (2018). Rheb1 loss leads to increased hematopoietic stem cell proliferation and myeloid-biased differentiation in vivo. *Haematologica* 104 245–255. 10.3324/haematol.2018.194811 30262562 PMC6355497

[B26] Wang X. Li M. Gao Y. Gao J. Yang W. Liang H. (2016b). Rheb1-mTORC1 maintains macrophage differentiation and phagocytosis in mice. *Exp. Cell Res.* 344 219–228. 10.1016/j.yexcr.2016.04.017 27163399

[B27] Zhu H. Guo Z. K. Jiang X. X. Li H. Wang X. Y. Yao H. Y. (2010). A protocol for isolation and culture of mesenchymal stem cells from mouse compact bone. *Nat. Protoc.* 5 550–560. 10.1038/nprot.2009.238 20203670

[B28] Zou J. Zhou L. Du X. X. Ji Y. Xu J. Tian J. (2011). Rheb1 is required for mTORC1 and myelination in postnatal brain development. *Dev. Cell* 20 97–108. 10.1016/j.devcel.2010.11.020 21238928 PMC3056331

