# Supplementary material for: Rheb1-Deficient Neutrophils Promote Hematopoietic Stem/Progenitor Cell Proliferation via Mesenchymal Stem Cells
Source: Front Cell Dev Biol. 2021 May 27;9:650599. doi: 10.3389/fcell.2021.650599 (PMC8191467; doi:10.3389/fcell.2021.650599)
Supplement: Supplementary file 1 [file Table_1.DOCX]

| **Supplementary table 1** | | | | | |
| --- | --- | --- | --- | --- | --- |
| Antibody | Clone detail | | Cat number | Company | Concentration |
| anti-mouse CD3 biotin | 145-2C11 | 2023-x26 | | \| eBioscience \| \| --- \| \|  \| \|  \| | 0.5 mg/ml |
| anti-mouse CD4 biotin | RM4-4 | 13-0043-85 | | eBioscience | 0.5 mg/ml |
| anti-mouse CD8a biotin | 53-6.7 | 13-0081-85 | | eBioscience | 0.5 mg/ml |
| anti-mouse CD11b BIOTIN | M1/70 | 13-0112-85 | | eBioscience | 0.5 mg/ml |
| anti-mouse CD45R(B220) biotin | RA3-6B2 | 13-0452-85 | | eBioscience | 0.5 mg/ml |
| anti-mouse TER-119 biotin | TER-119 | 13-5921-85 | | Invitrogen | 0.5 mg/ml |
| anti-mouse CD117(c-Kit) APC | 2B8 | 17-1171-82 | | Invitrogen | 0.2 mg/ml |
| anti-mouse Ly-6G(Gr-1) PE-Cyanine7 | RB6-8C5 | 25-5931-82 | | Invitrogen | 0.2 mg/ml |
| anti-mouse Ly-6A/E (Sca-1) PE-Cyanine7 | D7 | 558162 | | eBioscience | 0.2 mg/ml |
| anti-mouse CD11b APC | M1/70 | 17-0112-83 | | eBioscience | 0.2 mg/ml |
| anti-mouse CD34 FITC | RAM34 | 11-0341-85 | | Invitrogen | 0.5 mg/ml |
| anti-mouse CD45.2 PE | 104 | 12-0454-81 | | Invitrogen | 0.2 mg/ml |
| anti-mouse CD45.1 Percp-Cy5.5 | A20 | 45-0453-82 | | Invitrogen | 0.2 mg/ml |
| Streptavidin APC-Cy7 |  | 47-4317-82 | | Invitrogen | 0.2 mg/ml |
| anti-mouse CD45.1 FITC | A20 | 11-0453-85 | | eBioscience | 0.5 mg/ml |
| APC RAT anti-mouse CD31 APC | MEC-13.3 | 551262 | | eBioscience | 0.2 mg/ml |
| anti-mouse CD115(c-fms）PE | AFS98 | 12-1152-82 | | Invitrogen | 0.2 mg/ml |
| anti-mouse CD51 PE | RMV-7 | 12-0512-82 | | eBioscience | 0.2 mg/ml |
| anti-mouse F4/80 FITC | BM8 | 11-4801-82 | | Invitrogen | 0.5 mg/ml |
