# Supplementary material for: Rheb1-Deficient Neutrophils Promote Hematopoietic Stem/Progenitor Cell Proliferation via Mesenchymal Stem Cells
Source: Front Cell Dev Biol. 2021 May 27;9:650599. doi: 10.3389/fcell.2021.650599 (PMC8191467; doi:10.3389/fcell.2021.650599)
Supplement: Supplementary file 2 [file Data_Sheet_1.DOCX]

**
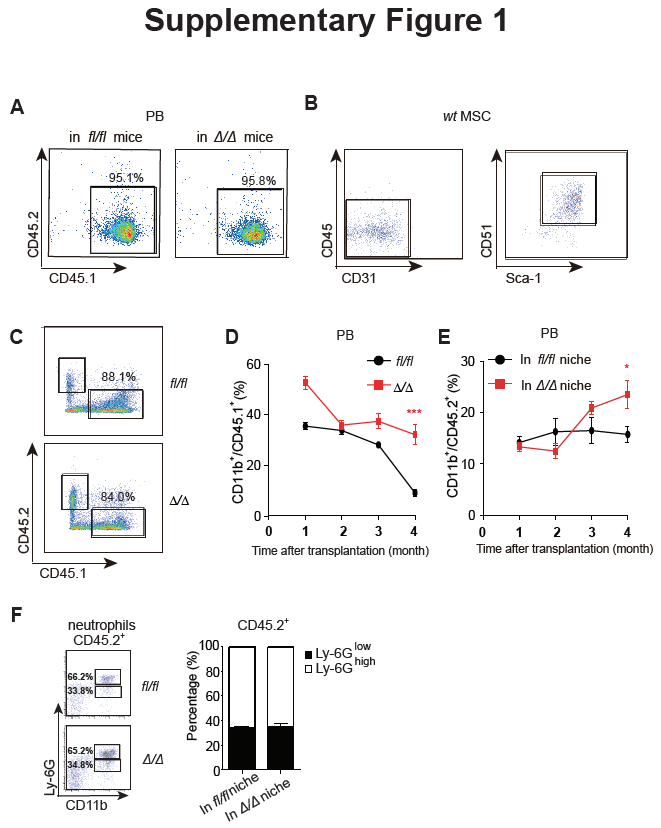
**

**Supplementary Figure 1. *WT* HSCs could be differentiated into normal neutrophils in the presence of *Rheb1^Δ/Δ^* blood cells in bone marrow.**

(A) The percentage of CD45.1^+^ cells in PB of *Rheb1^fl/fl^* and *Rheb1^Δ/Δ^* mice. (B) The surface markers of isolated MSCs from bone of mice. (C) The percentages of CD45.1^+^ cells and CD45.2^+^ cells in BM four months after transplantation. (D)The percentage of donor-derived CD11b^+^ cells (CD45.1^+^) in PB. (E) The percentage of donor-derived CD11b^+^ cells (CD45.2^+^) in PB. (F) The percentage of donor-derived CD11b^+^Ly-6G^high/low^ cells (CD45.2^+^) in BM four months after transplantation. The data are presented as the mean ± SD, n=3.

**
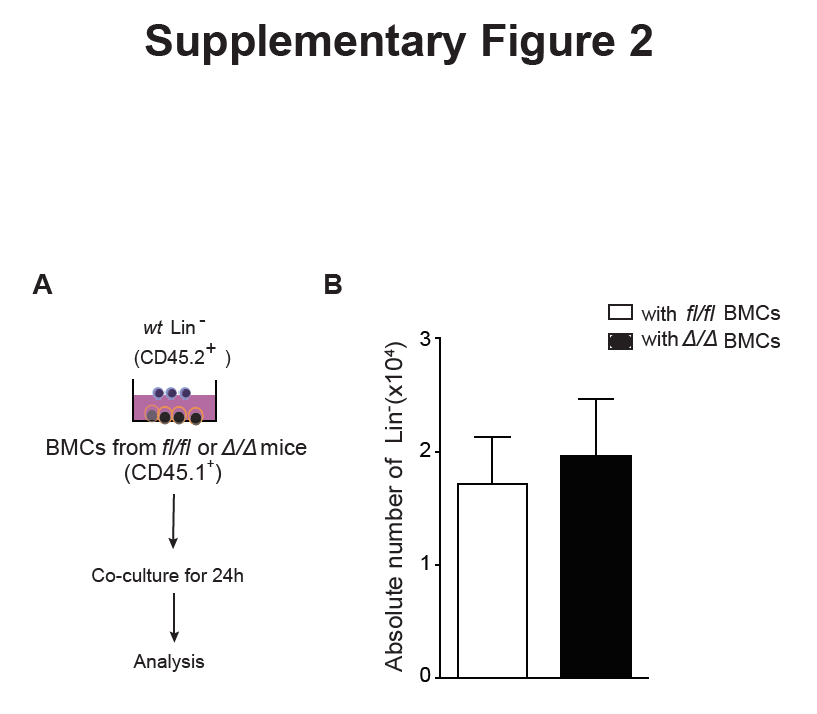
**

**Supplementary Figure 2. *Rheb1^Δ/Δ^* blood cells did not promote HSC proliferation *in vitro*.**

1. *WT* Lin^-^ cells (CD45.2^+^) with BMCs from *Rheb1**^fl/fl^* and *Rheb1^Δ/Δ^* mice (CD45.1^+^) cocultured for 24 h. (B) The absolute number of Lin^-^ cells after cocultured with either *Rheb1 ^fl/fl^* or *Rheb1^Δ/Δ^* BMCs for 24 h. The data are presented as the mean ± SD, n=3.


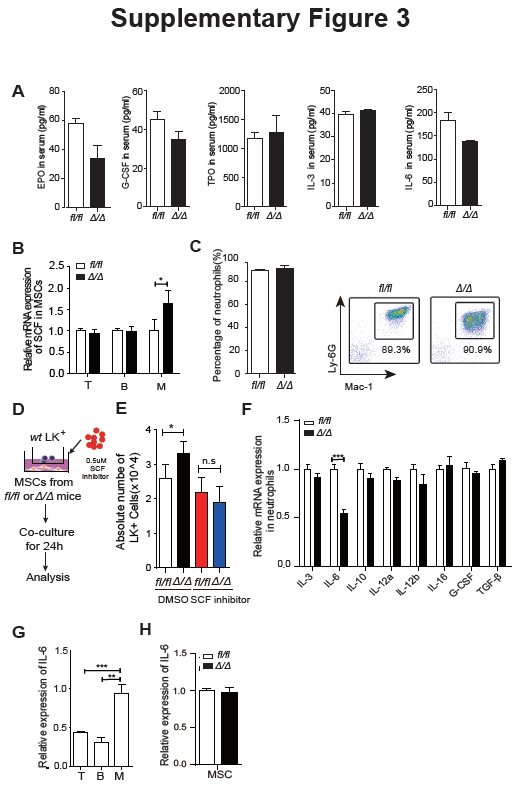


**Supplementary Figure 3. The expression of IL-6 and other cytokines in blood cells in *Rheb1^fl/fl^* or *Rheb1^Δ/Δ^* mice.**

(A) The protein level of EPO, G-SCF, TPO, IL-3 and IL-6 in the serum of *Rheb1^fl/fl^* and *Rheb1^Δ/Δ^* mice. (B) The relative mRNA expression of SCF in *wt* MSCs cocultured with *Rheb1^fl/fl^* or *Rheb1^Δ/Δ^* T cells, B cells and myeloid cells for 12 h. (C) Left panel, the percentage of isolated neutrophils from BM of *Rheb1^fl/fl^* and *Rheb1^Δ/Δ^* mice. Right panel, the surface markers of isolated neutrophils from BM of *Rheb1^fl/fl^* and *Rheb1^Δ/Δ^* mice. (D-E) The number of LK^+^ cells cocultured with MSCs from *Rheb1^fl/fl^* and *Rheb1^Δ/Δ^* mice for 24 h after adding SCF inhibitor (0.5 mM). (F) The relative mRNA expression of selected genes in *Rheb1^fl/fl^* or *Rheb1^Δ/Δ^* neutrophils isolated from BM. (G) The relative mRNA expression of IL-6 in T cells, B cells and myeloid cells from *wt* mice. (H) The relative mRNA expression of IL-6 in MSCs from *Rheb1^fl/fl^* or *Rheb1^Δ/Δ^* mice. The data are presented as the mean ± SD, n=3.
